# Topics of Public Interest

**Published:** 1911-08

**Authors:** 


### TOPICS OF PUBLIC INTEREST

THE British Medical Committee that has been studying spiritual healings for ten years, has reported that it finds no essential difference between this mode of treatment and mental suggestion in general.

THE North American Children's Sanitarium for the Treatment of Surgical Tuberculosis, has recently been opened at Atlantic City. Drs. H. Augustus Wilson, John B. Carnett and Wm. L. Rodman of Philadelphia are the executive committee; Dr. John C. Tull, the resident surgeon; the consulting staff embracing a long list of physicians and surgeons, mainly of Philadelphia and Atlantic City. This charity is to continue the good work of the N. A. Sanitarium for Convalescent Children which, last year, cared for over 1000 patients. The only restrictions are that patients must be white and under 12 years of age, admission being in order of date of application, from a waiting list.

MAYOR FUHRMANN has signed the bill authorising the city to establish a hospital and industrial colony for inebriates. This bill has been supported by the Charity Organisation Society and aims to make possible the scientific and humane treatment of drunkards.

The Health Department, the District Nursing Association, the Society for the Relief and Control of Tuberculosis and the Charity Organisation Society, have appointed a joint committee to carry on the crusade against the "White Plague" in Buffalo. Weekly conferences will be held.

### Post-Operative Hemorrhage

WHIPPLE and Hurwitz, *Jour. Exp. Med.,* Vol. 13, No. 1, in investigating the action of chloroform on the liver, noted that several dogs died of uncontrollable hemorrhage. This might be due to any of three deficits—calcium, thrombin or fibrinogen. Calcium was found by analysis not to be deficient. Thrombin was shown to be present in normal amount by the fact that the blood actually coagulated with normal rapidity—in 4—7 minutes after withdrawal—but the clot was lacking in tenacity and small in volume. Hence, the fault was obviously due to lack of fibrinogen.

Fibrinogen is either formed within the liver or, at any rate is due to hepatic activity and its failure was shown to be due to and to correspond in degree to hepatic necrosis. It was usually necessary to administer chloroform for two hours to produce marked hepatic necrosis and, even when the necrosis, which begins in the center of the lobule, involved half or three-fifths of the lobule, repair usually begins on the second and third day and is practically complete in a week.

Thus, we may conclude that while this study is extremely interesting, post-operative hemorrhage is not to be expected after chloroform anesthesia of any reasonable duration in patients free from hepatic disease.

---

Leslie Roberts (*Brit. Jour. Derm.* June, 1911), reports the death of a woman aged 21, from Acute Lupus Erythematosus (Aigu D'Emblée.)

The origin of the affection was not determined but on the father's side there was a marked history of tuberculosis (seven of a family of eight having died from pulmonary and laryngeal phthisis) and the post mortem examination in this case showed the mesenteric glands to be the seat of an old caseous tuberculosis.

This woman, who had enjoyed good health, suddenly complained of extreme lassitude, sore throat and difficulty in swallowing which induced her physician to make a diagnosis of quinsy. Five months later she complained of severe pains in her arms and legs. One month later an eruption appeared on the cheeks which increased week by week until the face was swollen and suffused with a full, deep red color symmetrically distributed over the nose, cheeks, eyelids and ears. On the cheeks were dark greenish-yellow crusts beneath which the skin was ulcerated; purpuric petechiae were scattered over the chest, abdomen and upper and lower limbs; erythematous patches appeared on the palmar surfaces of the fingers and the left great toe. The symptoms steadily increased in severity, a septic temperature set in and the patient died eight months from the first appearance of the disease.

Attention is called to the sex of the patient, the mysterious origin, vague early symptoms, rheumatoid muscular pains and painful spots in various parts, increasing weakness and loss of appetite. The early eruption may simulate erysipelas; later it may exhibit an eczematous appearance. Purpura and hemorrhage are fairly constant. Prognosis is bad; of eleven cases so far reported, ten have died.

Roberts concludes that Lupus Erythematosus is probably the excretion of a toxin by limited areas of skin followed by a cytolytic and erythematous reaction and its frequent association with adenitis seems to warrant the hypothesis that the disease is caused by a cytolytic poison which originates in the leukocytes of the lymph glands. W. W. Q.

